# Human NK Cell Subsets in Pregnancy and Disease: Toward a New Biological Complexity

**DOI:** 10.3389/fimmu.2016.00656

**Published:** 2016-12-27

**Authors:** Costanza Maria Cristiani, Eleonora Palella, Rosa Sottile, Rossana Tallerico, Cinzia Garofalo, Ennio Carbone

**Affiliations:** ^1^Department of Experimental and Clinical Medicine, University Magna Graecia of Catanzaro, Catanzaro, Italy; ^2^Department of Microbiology, Cell and Tumor Biology, Karolinska Institutet, Stockholm, Sweden

**Keywords:** NK cells, chemokines, melanoma, anatomical distribution, diagnosis

## Abstract

In humans, NK cells are mainly identified by the surface expression levels of CD56 and CD16, which differentiate between five functionally different NK cell subsets. However, nowadays NK cells are considered as a more heterogeneous population formed by various subsets differing in function, surface phenotype, and anatomic localization. In human CMV- and hantaviruses-infected subjects, an increased frequency of a NKG2A^−^CD57^+^NKG2C^+^ NK cell subset has been observed, while the phenotype of the NK cell subpopulation associated with cancer may vary according to the specific kind of tumor and its anatomical location. The healthy human lymph nodes contain mainly the CD56^bright^ NK cell subset while in melanoma metastatic lymph nodes the CD56^dim^CD57^+^KIR^+^CCR7^+^ NK cell subpopulation prevails. The five NK cell subpopulations are found in breast cancer patients, where they differ for expression pattern of chemokine receptors, maturation stage, functional capabilities. In pregnancy, uterine NK cells show a prevalence of the CD56^bright^CD16^−^ NK cell compartment, whose activity is influenced by KIRs repertoire. This NK cell subset’s super specialization could be explained by (i) the expansion of single mature CD56^dim^ clones, (ii) the recruitment and maturation of CD56^bright^ NK cells through specific stimuli, and (iii) the *in situ* development of tumor-resident NK cells from tissue-resident CD56^bright^ NK cells independently of the circulating NK cell compartment. This new and unexpected biological feature of the NK cell compartment could be an important source of new biomarkers to improve patients’ diagnosis.

## Introduction

Innate lymphoid cells (ILCs) are a family of mononuclear hematopoietic cells participating to tissue immunity (1). Natural killer (NK) cells are prototypic members of the ILCs group 1. They are characterized by the capability to recognize and kill virus-infected or transformed target cells through a balance of activating and inhibitory signals given by different surface receptors, in the absence of antigen-specific priming (2). In humans, NK cells are identified by the surface expression of CD56 and the lack of CD3 (3). One of the first well-defined NK cell activating receptors is CD16, the low-affinity receptor for FcγRIII responsible for Ab-dependent cellular cytotoxicity (4). CD56 and CD16 expression levels discriminate between five NK cell subpopulations. CD56^dim^CD16^+^ NK cells predominate in blood, at sites of inflammation, and show a high cytotoxic potential and broadly express MHC-I-specific inhibitory receptors. The CD56^bright^CD16^−^ and CD56^bright^CD16^+^ NK cell subsets predominate in lymph nodes, produce cytokines upon activation, express only one MHC class I-specific inhibitory receptor (NKG2A), display a low cytotoxic potential, and are considered to be a precursor of the terminally differentiated CD56^dim^CD16^+^ NK cells (5). CD56^bright^CD16^+^ and CD56^−^CD16^+^ NK cells are minor NK cell subpopulations whose role is largely unknown (6).

Today, based on receptor repertoire and expression levels, NK cells are considered as a more heterogeneous population formed by various subsets differing in function, surface phenotype, and anatomic localization. These different NK cell subpopulations are involved both in regulating physiological conditions and in the response against viral and tumor disease. Here, we review the main human NK cell subsets discovered in the context of infection, cancer, and pregnancy.

## Natural Killer Cell Subsets in Human Cytomegalovirus (CMV) Infection

As revealed by murine models, NK cells are essential for an effective defense against the herpes virus family and in particular against human and murine CMV (7). The co-evolution of the CMV and our species’ immune system is evident considering the virus escape strategies. Human CMV (HCMV) inhibits the expression of human leukocyte antigen (HLA) class I molecules, interfering with antigen presentation to CD8^+^ T cells (8). HCMV genome encodes for two early genes, US2 and US11, that directly interact with the nascent MHC-I, re-routing the molecules from the endoplasmic reticulum to the proteosomal compartment, thus preventing the correct expression of MHC-I on the infected cells surface (9). On the other side, HLA class I molecules loss in HCMV-infected cells reduces the engagement of inhibitory receptors, prompting NK cell effector functions (10). However, HMCV expresses two late genes, UL40 and UL18, that compensate the loss of classical MHC-I molecules overexpressing non-classical MHC-I (HLA-E) (11) or directly recruiting the NK cell inhibitory receptor ITL-2, respectively (12).

The above described viral escape strategy is counteracted by our immune system due to the expansion of a specific NK cell subset that actively recognizes the molecular signature of the HCMV escape strategy. Indeed, the main surface marker characterizing a specific NK cell subset in the context of HCMV infection is the activating receptor NKG2C, a C-type lectin covalently assembled with CD94 that specifically recognizes HLA-E molecules (13). Studies *in vitro* revealed that the interaction between peripheral blood NK cells and HCMV-infected fibroblasts induces the preferential proliferation of NKG2C^+^ NK cell subset through the direct involvement of the CD94/NKG2C receptor (14). A higher proportion of NKG2C^+^ NK cells after HCMV infection have been further observed in children with symptomatic congenital HCMV infection (15) and in HCMV^+^ healthy adults. In this latter case, NKG2C^+^ NK cells preferentially co-express CD57, a surface marker for highly mature NK cells, while they do not express NKG2A, the inhibitory counterpart of NKG2C. Therefore, these NK cells are a unique population of NKG2A^.^CD57^+^NKG2C^+^ NK cell clones that are absent in HCMV-seronegative donors (16).

Analyses performed on solid-organ transplanted (SOT) recipients with acute HCMV infection clarified the development of this subset in several discrete steps marked by the acquisition on the NK cell surface of a specific set of receptors: (a) increase of NKG2C amount, (b) acquisition of CD57 expression, and (c) increase of CD57 expression, resulting in the terminal full mature subset phenotype CD57^+^NKG2C^bright^ HMCV-associated NK cell subset (17). The mechanism by which this NK cell subset interacts with HCMV-infected fibroblast has been modeled *in vitro* and seems to involve the cell adhesion molecule CD2, a co-activating receptor on NK cells, and its ligand CD58. Indeed, the molecular interference of the CD2–CD58 interaction results in a decreased activation of CD57^+^NKG2C^+^ NK cells with a reduced secretion of TNFα and IFNγ (18).

A similar increase in NKG2C^+^ NK cells was observed in hematopoietic cell transplantation (HCT) recipients who reactivate HCMV after transplantation. In this context, it has been shown that the NKG2A^.^CD57^+^NKG2C^+^ NK cells are also equipped with the killer cell immunoglobulin-like receptors (KIRs), which specifically recognize different HLA class I molecules. This latter immune phenotype feature is associated with a potent IFNγ secretory activity. This indicates that HCMV reactivation after HCT results in the expansion of a more mature and educated NK cell subset: NKG2A^-^KIRs^+^CD57^+^NKG2C^+^ NK cells. In addition, during HCMV reactivation in HCT recipients, NKG2C^+^ expanding NK cells predominantly express KIR2DL3 (19). This NK cell repertoire feature is shared also by HCMV^+^ chronic hepatitis patients, where the KIR expressed on NKG2C^+^ NK cells is in most cases specific for self-HLA class I ligands, making the anti-virus specific NK cell subset able to discriminate between HLA-I self virus-infected and healthy cells (20). Moreover, in heart- and lung-transplanted patients, upon HCMV either reactivation or infection, an increased frequency of the NK cell subset expressing the inhibitory receptor LIR-1 recognizing the MHC class I homolog UL18 has been observed (21).

In HCMV^+^ healthy subjects, the activating KIRs (KIR2DS2, KIR2DS4, and KIR3DS1) also play a role in the adaptation of the NK cell compartment to HCMV infection. This activating receptor clusters mark a highly differentiated NK cell subset present in the periphery of HCMV^+^ healthy subjects regardless of NKG2C expression (22). The appearance and expansion of these NK cell subpopulations seem to be HCMV-specific, since the two phenomena are not induced by other human herpes viruses such as Epstein–Barr virus (22, 23).

A recent study demonstrated that HCMV infection was also related to a distinct subset of NK cells characterized by a deficiency in the expression of FcRγ (also known as FcεRIγ), associated with high amounts of NKG2C and low levels of natural cytotoxicity receptors NKp30 and NKp46. It is conceivable that this finding could be an effect of the HCMV infection. From a functional point of view, this NK cell subset responds poorly to HCMV-infected cells directly, yet it increases its efficiency against infected target cells in the presence of HCMV-specific IgG. FcRγ deficiency and the associated phenotype seemed to be due to a down-modulation of the tyrosine kinase SYK, stably maintained through the hypermethylation of a specific region in the SYK promoter DNA sequences (24).

## Natural Killer Cell Subsets in Hantaviruses Infection

Pathogenic hantaviruses (HTNV) are zoonotic viruses that cause hemorrhagic fever with renal syndrome or hantavirus pulmonary syndrome in humans (25). Both syndromes are characterized by vascular permeability and increased immune activation, with strong cytotoxic lymphocyte expansion of highly mature NKG2A^−^CD57^+^NKG2C^+^CD56^dim^ NK cells (26). A deeper analysis of this subset showed that it also expresses high levels of the early activation marker CD69, the activating NK cell receptors NKG2D and 2B4 and NCRs. This activated phenotype has been proposed to be dependent on the increased surface expression of IL-15 and IL-15Ra on hantavirus-infected endothelial and epithelial cells. On the other hand, HTNV infection weakens the effector responses of activated NK cells by increasing HLA class I expression on endothelial cells (27).

## Natural Killer Cell Subsets in Cancer

Although NK cells recognition and killing of tumor cells has been widely demonstrated *in vitro*, their *in vivo* activity against tumors remains less known. Generally, the presence of tumor-infiltrating NK cells indicates an ongoing immune response against the tumor and a better prognosis; however, some reports suggest that it can not correlate with the prognosis or be associated with advanced disease (28). Therefore, to appreciate the real prognostic value of the tumor NK cell infiltrate more investigations are required.

Nowadays, phenotypically distinct NK cell populations have been identified in different tissues, where they could represent specialized subsets mediating different functions (5). The identification and characterization of various NK cell subsets represents, therefore, an important advance in the understanding of tumors biology and could have deep implications in the monitoring and treatment of the disease.

In non-small cell lung cancer (NSCLC) patients, tumor-infiltrating natural killer (TINK) cells show a very peculiar surface molecular pattern, with high levels of surface CD56 and KIRs but lack of expression of CD16. Based on its perforin content, this CD56^bright^CD16^−^KIRs^+^ NK cell subset seems to derive from peripheral CD56^bright^CD16^−^KIR^−^ NK cells, suggesting that CD56^bright^CD16^−^ NK cells can acquire KIRs expression within tumor tissues as a consequence of signals from tumor microenvironment (29). Another study demonstrated that TINK cells from NSCLC patients exhibit a significant reduction in CD11b and CD27 expression, two markers mainly used to distinguish murine NK cell subpopulations. This CD11b^−^CD27^−^ subset is also characterized by the downregulation of CD57, CD16, DNAM-1, and NKp30, while CD127 and CD117 are clearly expressed, and NKG2A is slightly upregulated, suggesting an immature and/or inactive phenotype of these TINK cells (30).

In breast cancer patients, blood NK cells show strong alterations that became more dramatic with the advance of the disease. Blood NK cell compartment display a reduction in activating receptors (NKp30, DNAM-1, NKG2D, 2B4, CD16) and an upregulation of the inhibitory receptors NKG2A and ILT-2, which are functionally related with defective degranulation and ADCC-mediated killing capabilities. These phenotypic and functional changes are more evident in tumor-infiltrating NK cells, in which a prevalence of the CD56^bright^ compartment has been observed (31). Peripheral NK compartment has been further characterized distinguishing for the five subpopulations, whose proportions varying according to the disease stage. Most CD16^+^ NK cells show different combinations of KIRs except KIR2DL4, that in contrast is expressed on the CD56^dim^CD16^−^ NK cell subpopulation. The CD56^bright^CD16^−^ NK cell subset lacks the inhibitory KIRs and display low frequencies of KIR2DL4 and KIR2DS4, possibly indicating a reduced functional ability. CD57 is strongly expressed on CD56^dim^CD16^+^ and CD56^bright^CD16^+^ NK cells, while is downregulated in the others three subsets, suggesting that they are less mature and differentiated. CD57 expression also correlates with perforin and granzyme B levels, reflecting the cytotoxic potential of the different NK cell subsets. The five NK cell subsets show also difference in the expression pattern of chemokine receptors. CD56^dim^CD16^+^ NK cells express CX3CR1 and CXCR1 receptors, in accordance with their ability to reach inflamed tissues. CD56^dim^CD16^−^ NK cells are characterized by CXCR1, CXCR4, and CD62L expression, suggesting a preferential tropism for both inflamed tissues and lymph nodes. CD56^bright^CD16^−^ and CD56^bright^CD16^+^ NK cell subsets display similar patterns, with high levels of CCR7, CD117, and CD62L, underlying a favorite tropism for secondary lymphoid organs. Functionally, CD56^dim^CD16^+^ NK cells have the strongest cytotoxic with a high degranulation potential. Conversely, CD56^dim^CD16^−^ and CD56^bright^CD16^−^ NK cell subsets are poorly cytotoxic, producing low amounts IFNγ and TNFα, while the CD56^bright^CD16^+^ NK cell subpopulation behaves as an intermediary between CD56^bright^CD16^−^ and CD56^dim^CD16^+^ NK cells (32).

An increase of NK cell frequency has been observed within the tumor-invaded lymph nodes (TILNs) of advanced stages melanoma patients, with a prevalence of the CD56^dim^ NK cell subpopulation characterized by a high expression of CD57, KIRs, CD69, and the homing chemokine receptor CCR7, suggesting overall a more differentiated effector phenotype actively migrating in the TILNs. Furthermore, peripheral blood CD56^dim^ NK cell subset of these patients has an increased expression of the chemokine receptor CXCR2 recognizing interleukin-8, one of the chemokines found in TILNs (33). TILNs from melanoma patients also contain a peculiar CD56^bright^ NK cell subset co-expressing CD16. This NK cell subpopulation has high percentages of NKp46^+^, NKG2D^+^, and KIRs^+^ NK cells and increased levels of perforin, suggesting a more differentiated CD56^bright^ NK cell subset that may exert antitumor activities (34).

Recently, a PD-1^+^ NK cell subpopulation has been found in peripheral blood and ascites from seropapillary ovarian carcinoma patients. PD-1^+^ NK cells are confined to the CD56^dim^ NK cell subset and are primarily composed by NKG2A^−^KIRs^+^LIR-1^+^CD57^+^ NK cells with low levels of NCRs and high expression of CD16 and perforin/granzyme, indicating that PD-1 expression is confined to terminally differentiated NK cells. However, PD-1^+^ NK cells show a low cytolytic activity against tumor targets cells and are poor cytokines producers (35).

## Natural Killer Cell Subsets in Pregnancy

The immunological features of the placentation and pregnancy is another research field where the expansion of specific NK subsets has been appreciated to play a crucial role.

In the first trimester of pregnancy, NK cells represent the 70% of CD45^+^ decidual leukocytes together with CD8^+^, CD4^+^, γδ T cells, and dendritic cells. These cells are defined “uterine NK” (uNK, also called decidual or dNK) and play a role both in the success of pregnancy and in the early human immunity response during infections. uNK cells are predominant in non-pregnant endometrium, increasing and changing their morphology during the secretory phase of the menstrual cycle (36). Prolactin and endometrial-derived IL-15 promote uNK cells stimulation and differentiation. During early pregnancy, uNK cells infiltrate the trophoblast supporting the placentation process. uNK cells control the remodeling of uterine spiral arteries, increasing the contact area between maternal blood and trophoblast cells (37, 38). uNK cells are identified by a different phenotype and functional characteristics compared to peripheral blood NK cells (pNK); they show a CD56^bright^CD16^−^ phenotype and express a repertoire of activating and inhibitory receptors (NKRs) belonging to early differentiation stages of pNK cells (39). uNK cells secrete vascular endothelial growth factor C, angiopoietin 2 (ANG2), placental growth factor (PIGF), cytokines that are involved in angiogenesis, and that do not belong to pNK cytokine profile (40).

Trophoblast cells express MHC-I molecules HLA-C, HLA-E, and HLA-G. HLA-E has high affinity for inhibitory receptor CD94/NKG2A expressed by all uNK cells (41); HLA-C is recognized by KIRs receptor family whose expression is induced directly *in utero*, while HLA-G is bound by ILT-2, that is present on uNK cells (42), and by the activating KIR2DL4 (43). The role of uNK cells during pregnancy has not been completely understood, but the mouse is used as model due to the presence of uNK cells in the decidua and to the expression of MHC repertoire on trophoblast cells. When the MHC-I is bound to NK cell receptors, the uNK cells are inhibited and this interferes with fetal growth and remodeling of decidual spiral arteries (44). In murine pregnancy, only maternal MHC contributes to educate uNK cells to acquire functional properties, while the presence of paternal-inherited allogeneic MHC allele inhibits uNK cells with complications on both fetal growth and arterial remodeling (45). In human pregnancy, KIR repertoire is specific for each mother, and fetal HLA-C (group C1 or C2) is specific for maternal and paternal HLA-C contribution. The presence of maternal KIR AA haplotype, constituted by inhibitory KIRs genes only, is associated with pregnancy disorders but the risk of pre-eclampsia is increased when the fetus presents more C2 genes than the mother or when it carries a paternal HLA-C2 epitope. Instead, KIR genes that are protective are localized on Tel-B region of B haplotype, that contains the inhibitory KIR2DL5A and the activating KIR3DS1, KIR2DS5, and KIR2DS1. Among them, only KIR2DS1 binds to HLA-C, and its maternal frequency correlates with protection from pre-eclampsia (46).

KIR2DL4 has been proposed to favor reproduction through a different mechanism. This activating KIR is expressed in the endosomal compartment of uNK cells, where it binds and accumulates the soluble form of HLA-G secreted by extravillous trophoblasts. KIR2DL4 engagement has been shown to lead to the activation of DNA damage response signaling pathways, resulting in the reprograming of uNK cells to a senescent state characterized by a very active metabolism and secretion of an array of soluble mediators. This regulated and sustained secretory response would shape and remodel the local environment to increase vascularization required for a growing fetus (43).

It has been shown that uNK cells have the capability to control HCMV infection, and actually they acquire cytotoxic phenotype against HCMV-infected decidual fibroblasts *in vitro*, modulating their receptor repertoire. NKG2D and CD94/NKG2C have an important role in uNK cell response, also demonstrated by cytokine production. Moreover, uNK cells are also able to infiltrate HCMV-infected tissues and to generate an immunological synapse. Further investigations are needed to characterize the innate immune defense mechanisms in controlling viral response during pregnancy *in vivo* (47).

## Conclusion

The deep and new knowledge on the composition of the human NK cell compartment reveals a more complex landscape than that appreciated before. Here, we gave an overview of the NK cell subsets enter recruited in specific pathological and physiological conditions (Table 1). This NK cell subsets’ super specialization could be possibly explained at least in the following three ways:

(1) ontogenetically determined mature CD56^dim^ NK cell subsets, arising from the circulating or secondary lymphoid tissues NK cell population in humans and equipped with clonally distributed activating and inhibitory NK cell receptors repertoire, scrutinize our tissues continuously preserving our body homeostasis. When the single NK cell subset equipped with the appropriate receptor/s to recognize the pathological associated molecular pattern is called in action as effector or regulatory cells upon the pathological stimulation, it will expand as classically described in mouse upon MCMV infection (48), in a process reminiscent of the adoptive immune memory.
(2) CD56^bright^ immature NK cells are recruited in the lymph nodes, other secondary lymphoid organs or inflammated tissues where they are exposed to stimuli that induce their activation and maturation, i.e., cytokines, cell-to-cell interactions, and chemokine stimulation. The different combinations of biological stimuli will dictate the maturation toward a given subset.
(3) Tumor-resident NK cells could develop locally from tissue-resident CD56^bright^ NK cells independently of the circulating NK cell compartment, likely due to the organ and/or tumor microenvironment (49).

**Table 1:** **Markers characterizing NK cell subset in pathological and physiological contexts**.

Context	Anatomical localization	NK subpopulation	Upregulated markers	Downregulated markers	Reference
Human cytomegalovirus infection	Peripheral blood	CD56^dim^	NKG2C CD57 KIRs LIR-1	NKG2A FcRg NCRs	(14, 16, 17, 19–22, 24)

Human hantaviruses infection	Peripheral blood	CD56^dim^	NKG2C CD57 CD69 NKG2D 2B4 NCRs	NKG2A	(26, 27)

Non-small cell lung cancer	Tumor	CD56^bright^	NKG2A KIRs CD117 CD127	CD16 CD11b CD27 CD57 DNAM-1 NKp30	(30)

Breast cancer	Peripheral blood	CD56^bright^CD16^−^	CCR7 CD117 CD62L	KIRs CD57	(32)
	
		CD56^bright^CD16^+^	KIRs (except for KIR2DL4) CD57 CCR7 CD117 CD62L			

		CD56^dim^CD16^+^	KIRs (except for KIR2DL4) CD57 CX3CR1 CXCR1			

		CD56^dim^CD16^−^	KIR2DL4 CXCR1 CXCR4 CD62L	CD57		

Melanoma	Tumor-infiltrated lymph node	CD56^dim^	CD57 KIR2 CD69 CCR7		(33)

		CD56^bright^	CD16 KIRs NKp46 NKG2D		(34)

Ovarian carcinoma	Peripheral blood	CD56^dim^	PD-1	NKG2A	(35)

	Ascites		KIRs CD57 LIR-1 CD16	NCRs		

Pregnancy	Uterus	CD56^bright^	NKG2A KIRs ILT-2	CD16	(39, 41–43, 46).

Whatever the genesis of the repertoire of NK cell subsets in the health and pathology, we foresee in this new biological feature of the NK cell compartment a gold mine to obtain new biomarkers to improve patients’ diagnosis.

## Author Contributions

All the authors contributed in writing and revising the manuscript.

## Conflict of Interest Statement

The authors declare that the research was conducted in the absence of any commercial or financial relationships that could be construed as a potential conflict of interest.
